# Evaluating the expression profile and stability of different UCOE containing vector combinations in mAb-producing CHO cells

**DOI:** 10.1186/s12896-017-0330-0

**Published:** 2017-02-22

**Authors:** Fatemeh Nematpour, Fereidoun Mahboudi, Behrouz Vaziri, Vahid Khalaj, Samira Ahmadi, Maryam Ahmadi, Saedeh Ebadat, Fatemeh Davami

**Affiliations:** 1 Biotechnology Research Center, Pasteur Institute of Iran, Tehran, 1316943551 Iran; 2 Departments of Medical Biotechnology, Semnan University of Medical Sciences, Semnan, 3519899951 Iran

**Keywords:** Cell line development, Chinese hamster ovary (CHO), Monoclonal antibody (mAb), Ubiquitous chromatin opening elements (UCOE)

## Abstract

**Background:**

As the demand for monoclonal antibodies (mAb) increases, more efficient expression methods are required for their manufacturing process. Transcriptional gene silencing is a common phenomenon in recombinant cell lines which leads to expression reduction and instability. There are reports on improved antibody expression in ubiquitous chromatin opening element (UCOE) containing both heavy and light chain gene constructs. Here we investigate the impact of having these elements as part of the light chain, heavy chain or both genes during cell line development. In this regard, non-UCOE and UCOE vectors were constructed and stable Chinese hamster ovary (CHO) cell pools were generated by different vector combinations.

**Results:**

Expression analysis revealed that all UCOE cell pools had higher antibody yields compared to non-UCOE cells, Moreover the most optimal expression was obtained by cells containing just the UCOE on heavy chain. In terms of stability, it was shown that the high level of expression was kept consistence for more than four months in these cells whereas the expression titers were reduced in the other UCOE pools.

**Conclusions:**

In conclusion, UCOE significantly enhanced the level and stability of antibody expression and the use of this element with heavy chain provided more stable cell lines with higher production level.

## Background

Therapeutic recombinant monoclonal antibodies (mAbs) have become a major sector in the biopharmaceutical industry [1]. Currently, about fifty mAbs have been approved for the treatment of a variety of diseases which include cancer, autoimmunity, infectious diseases, and cardiovascular disorders [2, 3]. Improved methods and technologies need to be utilized, to meet the growing demand for mAbs [4]. Due to high structural complexity and sophisticated post transcriptional modification requirements, mammalian expression systems particularly CHO cell lines are the most preferred for mAb manufacturing [5]. However, generation of stable and high-yielding mammalian cell lines remain one of the most significant challenging issues facing researchers in the field of cell line development [5, 6].

Recent studies have indicated that this inefficiency is mainly caused by transcriptional silencing of heavy chain (HC) and light chain (LC) genes with no loss of recombinant gene copies [7–9]. DNA methylation especially at promoter CpG islands plays an important role in transgene transcription silencing [9–11]. It has been reported that the use of cis-acting epigenetic regulatory elements such as locus control regions (LCRs), matrix attachment regions (MARs) and ubiquitous chromatin opening elements (UCOEs) can protect transgenes from such adverse epigenetic events [12–15]. Among these anti-silencing elements, the incorporation of UCOEs into the expression vectors enhances the stability and expression level of transgenes in mammalian cells. UCOEs are methylation-free CpG Islands which are located within the promoter of ubiquitously expressed housekeeping genes. UCOE from the human HNRPA2B1-CBX3 locus (A2UCOE) has been used in combination with plasmid and lentiviral vectors for recombinant protein expression and gene therapy strategies, respectively [16–22].

Heterotetramer mAb molecules consist of two identical HC and two identical LC polypeptides. UCOE has already been exploited to improve mAb expression and stability, however it has been incorporated into both the heavy chain and light chain genes and the separate effect of this element on antibody chains remains to be evaluated [16, 21, 23]. To the best knowledge of the authors, this is the first study where the distinct effect of UCOE on H and L chains is assessed. To this end, non-UCOE and UCOE heavy and light chain vectors of a model antibody were constructed. Then stable CHO cell pools were developed by different vector combinations: non-UCOE vectors (CHO-HL), UCOE vectors (CHO-UHUL), UCOE heavy chain and non-UCOE light chain vectors (CHO-UHL) and non-UCOE heavy chain and UCOE light chain vectors (CHO-HUL). The expression vector sets used for the generation of CHO cell pools are summarized in Table 1. Finally, mAb production studies which include mRNA and protein expression levels, long-term stability and clonal cell line expression were compared between constructed pools. This study provides considerable applications in mammalian cell line development for more improved antibody expression.

**Table 1:** Summary of the expression vectors used for the generation of CHO cell pools

Cell pool name	Expression vector used for heavy chain	Expression vector used for light chain
CHO-HL	pTracer-CMV2- HC (pH)	pIRES2-DsRed2-LC (pL)
CHO-UHUL	pTracer-CMV2-UCOE-HC (pUH)	pIRES2-DsRed2-UCOE-LC (pUL)
CHO-UHL	pTracer-CMV2-UCOE-HC (pUH)	pIRES2-DsRed2-LC (pL)
CHO-HUL	pTracer-CMV2-HC (pH)	pIRES2-DsRed2-UCOE-LC (pUL)

## Methods

### Antibody expression vector construction

**Fig. 1:**
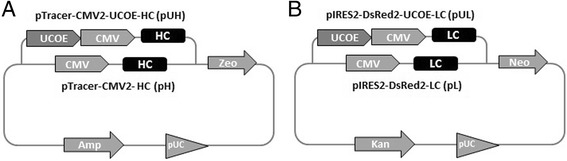
The schematic structure of plasmid vectors constructed and used in the studies. **a** Heavy chain coding plasmid vectors; pTracer-CMV2-HC (pH) and pTracer-CMV2-UCOE-HC (pUH). **b** Light chain coding plasmid vectors; pIRES2-DsRed2-LC (pL) and pIRES2-DsRed2-UCOE-LC (pUL)

A humanized IgG_1_ monoclonal antibody MAb0014 was chosen as the model antibody in this work. HC (gamma 1) and LC (kappa) cDNAs were commercially synthesized (Genscript) and cloned into separate expression plasmid vectors under the control of the same human cytomegalovirus (CMV) immediate-early enhancer and promoter. HC cDNA sequence was cloned into the pTracer-CMV2 vector (Invirtogen) which contained the zeocin resistance gene for selection of transfected cells. LC cDNA sequence was cloned into the pIRES2-DsRed2 vector (Clontech) with neomycin resistance gene. Heavy and light chain expression vectors were termed pH and pL, respectively. To construct UCOE containing vectors, synthesized 2.8 kb A2UCOE sequence (Genscript) was inserted into the upstream of CMV promoter of pH and pL vectors. These UCOE containing vectors were named pUH and pUL, respectively. The schematic maps of plasmid vectors used in this study are showed in Fig. 1. All vectors were constructed based on standard cloning methods [24]. The cloned sequences were confirmed by DNA sequencing.

### Cell culture

Suspension CHO DG44 host cell line (Life Technologies) (Catalog no: A10971-01) was used for antibody production. CHO cells were cultured in protein-free CD CHO medium (Life Technologies) supplemented with 8 mM L-glutamine (Life Technologies) and 1% penicillin/streptomycin (100 μg/mL) (Life Technologies) in disposable vented cap flasks and at 37 °C in humidified atmosphere of 5% CO_2_. Cells were routinely subcultured every three days at a density of 2–3 × 10^5^ cells/mL. Viable cell density was determined by using trypan blue (Sigma-Aldrich) exclusion method in duplicates [25].

### Transfection, stable cell pool generation and clonal isolation

Transfection was performed in duplicates in 6-well plates, 2 × 10^6^ cells were seeded in 2 mL CD CHO medium and transfected with 3 μg linearized plasmid DNA, using X-tremeGENE HP DNA transfection reagent (Roche) according to the manufacturer’s instructions.

To generate stable cell pools, CHO cells were transfected with LC vectors (pL or pUL) and selected with Geneticin (G418) (400 μg/mL) (Sigma-Aldrich) 72 h post-transfection. Cells were kept for about 4 weeks, and during this time period fresh medium with selection agent was added every three days until cell viability reach over 95%.

Thereafter, cells were transfected with HC vectors (pH or pUH) and selected with zeocin (500 μg/mL) (Life Technologies) 72 h post-transfection for 4 weeks. Finally, transfectants were cultured under dual G418 (400 μg/mL) and zeocin (500 μg/mL) selection pressure for up to 2 weeks. Four different cell pools were generated by transfection of pH and pL vectors (CHO-HL), pUH and pUL vectors (CHO-UHUL), pUH and pL vectors (CHO-UHL), and pH and pUL vectors (CHO-HUL). Following transfection and antibiotic selection steps, cell pools were passaged several times and transferred in duplicate into 6-well plates at a density of 5 × 10^5^ cells/mL for 7 days. Culture medium of stable cell pools were harvested at the end of batch culture and centrifuged at 4 °C 1100 rpm for 10 min. The supernatants were collected into a fresh 1,5 mL tubes and stored at −20 °C for further expression analysis by western blot and enzyme-linked immunosorbent assay (ELISA). Single-cell clones were isolated from cell pools using standard limiting dilution. In brief, cells were seeded in 96-well plates at a density of 1 cell per well in 200 μl of CHO medium (Life Technologies) with supplements in the absence of G418 or zeocin selection pressure. After 21 days, recovered clones were transferred into 24-well plates. For each cell pool, fifty clones were randomly scaled up into 12-well plates and 7 days later their supernatants were collected for antibody expression screening by ELISA and then three clones that had the highest mAb levels were selected for specific productivity analysis.

### Cell-specific productivity evaluation

To evaluate cell-specific productivity of selected clones, 3 × 10^5^ cells/mL were seeded in duplicate in CD CHO medium (Life Technologies) with supplements in 6-well plates for up to 7 days and sampled daily to measure viable cell density and antibody titer. The viable cell density was evaluated by trypan blue (Sigma-Aldrich) exclusion method and antibody concentration was measured using ELISA. Cell-specific productivity (qmAb; pg/cell/day) was determined by plotting antibody titer (μg/mL) against the integral of viable cells (IVC) for 2–4 days.

### Production stability study

In order to study the long-term stability of antibody production, duplicate samples of CHO cells were maintained in suspension culture in the absence of (G418) and zeocin selective pressure for over 4 months. Routinely, every 2 weeks, cells were sampled to measure antibody titer by ELISA. Cells were seeded at 5 × 10^5^ cells/mL density in 6-well plates and supernatants were collected 7 days later for antibody titer measurement.

### Antibody quantification by ELISA

The monoclonal antibody concentration was measured by capture ELISA. Multi-well strips (Thermo Scientific Nunc) were coated with 100 μl rabbit anti-human IgG Fc gamma capture antibody (Thermo Scientific Pierce) at 1:16000 dilution in coating buffer (50 mM NaHCO_3_, pH 9) and incubated at 4 °C overnight. Wells were blocked with 150 μl PBS containing 1% (w/v) bovine serum albumin for 1 h at 37 °C. Sample culture supernatants (diluted in PBS if necessary) were loaded into wells in triplicate and incubated for 1 h at 37 °C. Horseradish peroxidase (HRP) conjugated goat anti-human IgG detector antibody (Sigma-Aldrich) was added at 1:32000 dilution and incubated for 1 h at 37 °C. 100 μl tetramethyl-benzidine (TMB) peroxidase substrate (Sigma-Aldrich) was added and proceeded at RT in the dark for up to 30 min. Finally, 100 μl 1 N H_2_SO_4_ (Merk) was added to stop the reaction and absorbance was measured at 450 nm using a PowerWave XSTM (BioTek) microplate reader. In all the experiments, a standard curve was generated using serial dilutions of standard human IgG (Genscript) and untransfected cell culture medium was used as a negative control.

### Western blot analysis

Supernatant of stably transfected CHO pools were subjected to standard reducing 12% polyacrylamide gel SDS-PAGE. The separated samples were transferred onto nitrocellulose membrane (GE Healthcare) using the Trans-Blot SD semi-dry transfer cell (Bio-Rad). The membrane was washed with PBS supplemented with 0.025% [v/v] Tween 20 and blocked with 5% skimmed milk (Merk) in PBS at 4 °C overnight. Then stained with HRP conjugated goat anti-human IgG at 1:5000 in PBS (Sigma-Aldrich) at RT for 1 h and developed using 3,3ˈ-diaminobenzidin (DAB) peroxidase substrate (Sigma-Aldrich). Human IgG (Genscript) with known concentration and culture medium from untransfected cells were used as positive and negative control in every experiment, respectively.

### RNA extraction and cDNA preparation

Total RNA was extracted from 10^6^ cells using the TRIreagent (Sigma-Aldrich) following the manufacturer’s instruction. The isolated RNA was treated with RNase-free DNaseI (Thermo Scientific) to eliminate DNA contamination. The quantity and quality of RNA were determined using the Nanodrop 1000 spectrophotometer (Thermo Scientific). cDNA synthesis was performed using Transcriptor First Strand cDNA Synthesis Kit (Roche) according to the manufacturer’s instruction.

### Genomic DNA extraction

Genomic DNA was extracted from 10^6^ cells using DNA isolation kit (Roche) according to the manufacturer’s manual. DNA concentration and purity was measured using Nanodrop 1000 spectrophotometer (Thermo Scientific).

### mRNA level and gene copy number analysis

Quantitative real-time PCR (qRT-PCR) was used to determine the HC and LC mRNA levels, as well as the gene copy numbers. Specific HC, LC, GAPDH and β-actin primers were designed using the Primer Express software 3 (Applied Biosystems). The following primer sets were used: HC, forward 5′- CGACGGCTCCACAAACTATAATCC-3′; reverse 5′-TGCCAGTGACCGAAATAGTGAGAC-3′, LC, forward 5′-CAGAGTGTGGACTACGATGGAGAC-3′; reverse 5′-CGGAGCCTGAGAACCTGGATG-3′, GAPDH, forward 5′-CACTCTTCCACCTTTGATGCTG-3′; reverse 5′-GTCCACCACTCTGTTGCTGTAGC-3′, β-actin, forward 5′-AAGTGTGACGTCGACATCCGCAAAGAC-3′; reverse 5′-GGTTGACCTGGAAGGGCCCATCATG-3′. GAPDH and β-actin housekeeping genes were used as the internal control for normalizing RNA and DNA variation, respectively. Amplification was performed using a SYBR Green master mix (Applied Biosystems) in an ABI 7500 system (Applied Biosystems). The thermal profile for the real-time PCR was 95 °C for 5 min followed by 40 cycles at 95 °C for 15 s and 60 °C for 1 min. The specificity of amplification was confirmed by melt curve analysis following a thermal cycle as follows: 95 °C for 15 min, 60 °C for 30 min and 95 °C for 15 min. HC and LC cDNA-containing plasmid vectors were used as templates to generate standard curves. GAPDH and β-actin standard curves were generated using a chosen cDNA and DNA samples as templates, respectively. All standard dilutions, negative control and samples were assayed in triplicate. Relative quantification analysis was calculated using the Pfaffl method [26].

### Statistical analysis

Expression analysis data was statistically analyzed using the one-way ANOVA test in GraphPad Prism 6 software environment to detect significant differences in mAb production between the generated cell pools. For all statistical analyses a value of *p <*0*.*05 was the level of statistical significance.

## Results

### The effect of UCOE on antibody expression level in stable CHO cell pools

In order to evaluate the distinct impact of UCOE on HC and LC and find the optimal expression condition for antibody production in CHO cells, four stable pools were generated. The non-UCOE CHO-HL pool expressed both antibody chains without UCOE regulation and was used as a control. The CHO-UHUL pool expressed HC and LC under the UCOE control and was used as a conventional UCOE system. CHO-UHL and CHO-HUL expressed just HC and LC under the UCOE regulation, respectively.

**Fig. 2:**
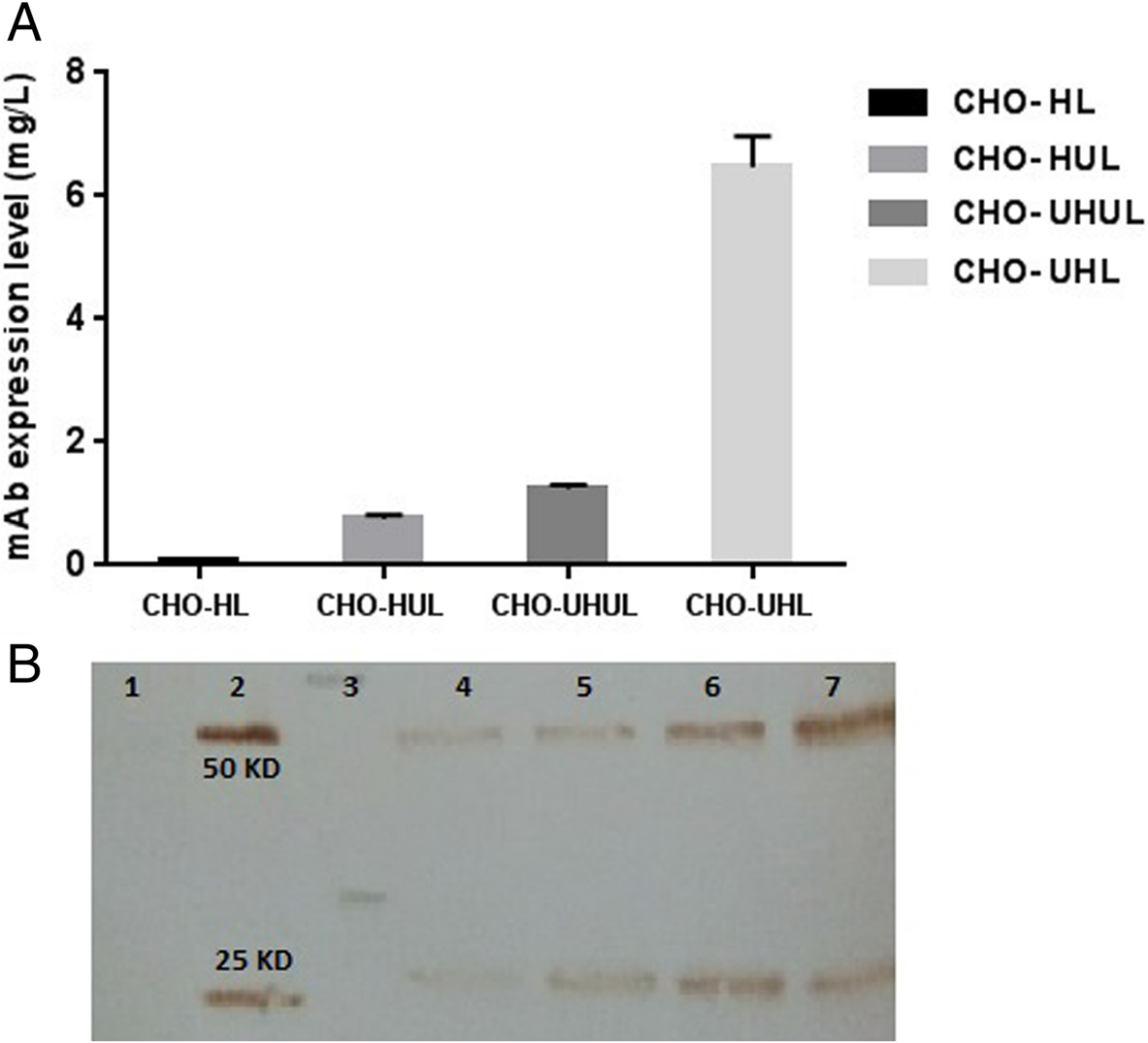
Antibody concentrations of different stable cell pools. **a** Antibody expression levels from generated pools were measured by ELISA. The error bars represent standard deviation of triplicate ELISA measurements. Data was statistically analyzed using ANOVA to detect significant differences between the generated cell pools (p < 0.05). **b** Western blot analysis of reduced supernatants from four pools using HRP conjugated goat anti-human IgG. The appearance of bands with the expected size of 50 KD for heavy chain and 25 KD for light chain verified the presence of antibody. Negative control (untransfected cell culture medium) (lane 1), Positive control (human IgG) (lane 2), protein molecular weight marker (lane 3), sample supernatants of CHO-HL (lane 4), CHO-HUL (lane 5), CHO-UHUL (lane 6) and CHO-UHL (lane 7)

Antibody expression level was compared between recovered pools, following the transfection of linearized plasmid vectors and selection with G418 (400 μg/mL) and zeocin (500 μg/mL). Duplicate samples for each cell pool were collected and measured by ELISA. The level of antibody expression is shown in Fig. 2a. In comparison with the non-UCOE control cell pool, all UCOE pools gave considerably higher antibody yields and different combinations of UCOE containing HC and LC constructs showed different expression levels. Analysis between UCOE pools showed that the expression level driven by CHO-UHL was greater than other UCOE cell pools. Expression level of CHO-UHL was 6.5 mg/L and about 8 times more than the CHO-HUL cells with 0.8 mg/L, and 5 folds higher than the conventional UCOE system (CHO-UHUL) with 1.2 mg/L. Moreover, Western blot analysis clearly verified the relative difference of antibody productivity between samples (Fig. 2b). In line with previous studies, these results indicate that the inclusion of UCOE on antibody chains provides a substantial improvement in CHO cell productivity [16, 21]. Based on the cell pools studied in the present work, it seems that utilization of this element on HC facilitates antibody expression.

### Effect of UCOE on antibody mRNA level and gene copy number

**Fig. 3:**
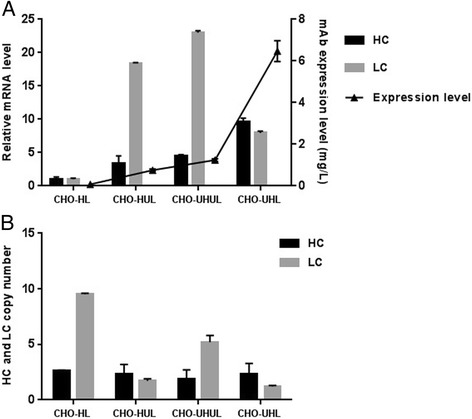
Antibody mRNA levels and gene copy numbers of the stable cell pools. **a** Relative HC, LC mRNA levels were determined by qRT-PCR and compared with antibody expression levels. **b** Relative HC, LC gene copy numbers were measured by qRT-PCR. The error bars represent standard deviation of three independent qRT-PCR assays. Data was statistically analyzed using ANOVA to detect significant differences between the generated cell pools (*p* < 0.05)

The HC and LC mRNA levels were measured by qRT-PCR (Fig. 3a). All UCOE pools showed higher HC and LC mRNA contents in association with their antibody titers, as compared to the non-UCOE pool. This suggests that UCOE, probably, increases productivity through enhancement of mRNA expression. The HC and LC mRNA levels of pools were also compared with their antibody titers (Fig. 3a). Subsequently, a positive correlation between HC mRNA level and the measured antibody concentration was found.

Genomic DNA of pools was analyzed by qRT-PCR to assess HC and LC gene copy numbers (Fig. 3b). As shown in Fig. 3b, the UCOE pools had almost about 2 and 3 copies of HC and LC, respectively, and control non-UCOE pool had 3 copies of HC and 10 copies of LC which indicated no apparent correlation between antibody production and copy number of recombinant genes.

### Evaluation of antibody expression of the stable clones

**Fig. 4:**
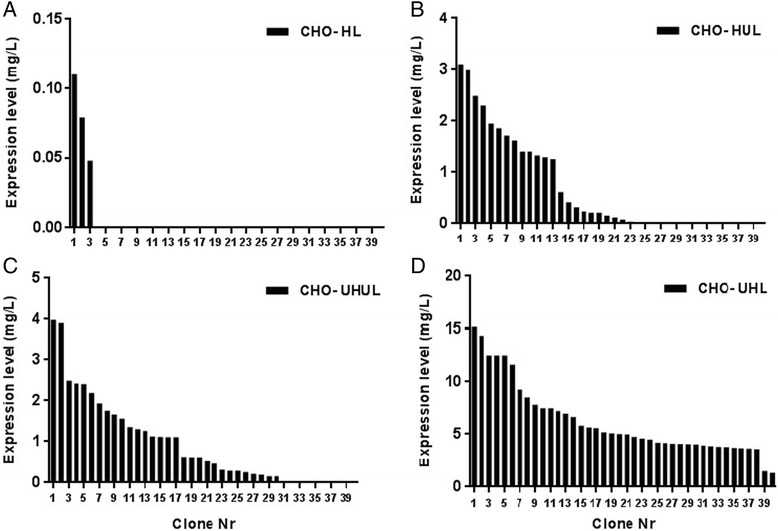
Antibody expression levels of clonal cell lines from CHO-HL **a** CHO-HUL (**b**), CHO-UHUL (**c**), CHO-UHL (**d**) pools are shown and ranked from highest to lowest. Data was statistically analyzed using ANOVA to detect significant differences between the generated cell pools (*p* < 0.05)

Stable clones were obtained by limiting dilution in the absence of antibiotic selective pressure. Forty random clones were picked and transferred into 24-well plates for each stably transfected pool. Antibody expression was measured by ELISA. Fig. 4a clearly shows that among clones derived from non-UCOE CHO-HL pool, only about 8% were able to express detectable level of antibody, while all UCOE containing cell pools showed significantly more positive antibody producing clones (Fig. 4b, c, d). CHO-UHUL and CHO-HUL pools had 80 and 60% positive clones, respectively. Interestingly, all clones from CHO-UHL pool produced antibody.

The average expression level detected in non-UCOE CHO-HL positive clones was about 0.08 mg/L, whereas it was about 1.2 mg/L in CHO-UHUL and CHO-HUL and 6.2 mg/L in CHO-UHL clones. These results demonstrate that UCOE could increase number of antibody expressing clones.

**Fig. 5:**
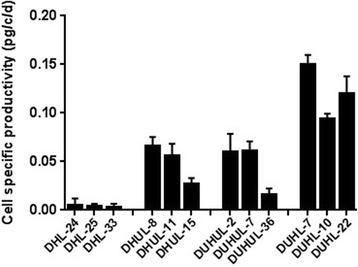
Comparison of specific antibody productivity (qmAb) [pg/cell/day] of three isolated clones from each cell pools. The error bars represent standard deviation of triplicate measurements. Data was statistically analyzed using ANOVA to detect significant differences between the generated cell pools (*p* < 0.05)

The best three expressing clones of each cell pool were selected to assess the cell-specific productivity. 3 × 10^5^ cells/mL were maintained in 24-well plates, and viable cell density and antibody titer were measured daily to calculate the specific productivity. The specific productivity of the clones obtained in the presence of UCOE was greater than that of the clones obtained in the absence of this element (Fig. 5). Comparison between the UCOE clones revealed that the average productivity of selected clones of CHO-UHUL and CHO-HUL was similar and isolated clones from CHO-UHL had the highest productivity.

### Effect of UCOE on antibody expression stability

**Fig. 6:**
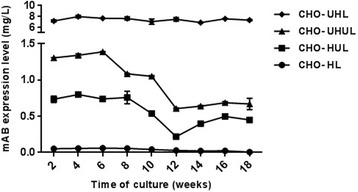
Long-term antibody expression levels of the stable cell pools. The error bars represent standard deviation of three experiment replicates Data was statistically analyzed using ANOVA to detect significant differences between the generated cell pools (*p* < 0.05)

In order to compare the long-term stability of antibody expression between generated stable pools, cells were cultivated in the absence of selective pressure for 4 months. Antibody titer was calculated every 2 weeks by ELISA. Generally, antibody levels from all UCOE containing cell pools remained higher than the non-UCOE cell pool over a long-term culture (Fig. 6). As shown in Fig. 6, expression level of the non-UCOE CHO-HL pool decreased much more rapidly by up to 90%, whereas it was slightly reduced by 50% for the CHO-UHUL and CHO-HUL pools. CHO-UHL maintained its expression level during this period. Consequently, in accordance with previous reports, UCOE gives higher production stability during long-term cultivation and based on the findings of the present work, it could be concluded that the addition of UCOE to HC not only maximizes antibody expression level but also provides more stable productivity [17].

## Discussion

To our best knowledge, previous studies used UCOE element in combination with both heavy and light chains for enhanced antibody production [16, 21, 23]. In this study, the individual effect of UCOE on HC and LC was investigated to find the optimal condition for antibody expression and stability.

At first, heavy and light chain encoding plasmid vectors were constructed. Then the UCOE containing versions of each vector were generated. The constructed vectors transfected into CHO-DG44 cells and four stable pools were developed: non-UCOE cell pool (CHO-HL), and three UCOE cell pools which one of them expressed both antibody chains under UCOE control (CHO-UHUL) and the two other cell pools expressed just HC or LC under UCOE regulation (CHO-UHL and CHO-HUL).

In agreement with other published works, it was found that UCOE containing pools had higher antibody titers when compared with the control cell pool lacking this element [16, 17, 21]. Additionally, expression analysis by ELISA and western blot showed the CHO-UHL pool, only expressing HC under the control of UCOE resulted in significantly higher antibody level than other UCOE pools. It was about 8- and 5-folds higher than the CHO-HUL and CHO-UHUL pools, respectively. The expression level of CHO-UHUL was about 1.5 times greater than that of CHO-HUL.

To find the possible reason for differences in antibody production, HC and LC mRNA expression and gene copy numbers were analyzed. It was observed that despite the lower copy number of HC and LC genes, all UCOE cell pools had greater mRNA levels. Hence, in agreement with previous data, it was shown that UCOE could exert its effect by enhancing transcription without any distinguishable increase in copy numbers of integrated transgenes [17, 27]. Comparison of HC and LC mRNA levels with antibody concentrations showed a direct correlation between antibody titers and heavy chain mRNA levels.

In addition, antibody expression analysis was performed on clonal cell lines. Among analyzed clones, UCOE containing pools showed more positive clones with higher antibody yields than the non-UCOE pool. This is consistent with the fact that more antibody secreting cell lines were obtained in the presence of UCOE [21, 28] probably due to its insulating effect on transgene expression. As expected, the number of positive clones and antibody levels were significantly greater in CHO-UHL and all colonies from this pool expressed mAb with an average of 6.2 mg/L which could indicate higher expression level for this pool. On the other hand, CHO-UHUL had more number of positive clones than CHO-HUL and the average antibody levels of both were approximately similar. These results suggested that expression up-regulation of antibody chains through UCOE could increase the number of antibody secreting clones and consequently improve productivity. What’s more, our findings indicated that up-regulation of HC expression may result in more positive clones and antibody production rates.

As observed in this work, UCOE enhances recombinant protein production in CHO cells by improvement in gene expression. In terms of monoclonal antibody the comparative study between generated UCOE containing cell pools indicated that the optimal antibody yields were obtained from CHO-UHL cells. Although, enhancement of LC expression appears to increase antibody production (CHO-HUL), improvement of HC expression has significantly more impact on secreted antibody levels (CHO-UHUL and CHO-UHL), and also the interesting point is that the enhancement of HC expression, has a better effect in comparison with enhancement of both HC and LC. In comparison with conventional CHO-UHUL system fortified HC expression by UCOE in CHO-UHL pool lead to more positive antibody secreting clones with higher specific productivity which finally resulted in higher antibody yield in this cell pool. In some previous publications it was indicated that expression of LC is more efficient than HC, and antibody production is limited by the expression of HC [29, 30]. Also, in some mRNA expression analysis it was reported that antibody productivity has a better correlation with HC rather than LC expression levels [30–32]. Therefore, because HC is a more limited component than LC, its expression enhancement may allow for better formation of the antibody. So it was deduced that in CHO-UHL cells HC is no longer limiting which caused a higher antibody production rate. In other words, considering the higher LC mRNA levels in CHO-UHUL compared to CHO-UHL, it seems that if the cell is in the situation that can produce both LC and HC mRNA in an epigenetic protected condition, LC transcription and expression would be dominant probably due to shorter length and simpler transcript. This, we assume that may deplete the energy sources towards LC rather than HC and finally the equal amount of HC and LC which is needed for proper final folding would not be achieved.

One of the major challenges in the industrial manufacturing of antibodies is transgene expression instability over the long-term culture. Epigenetic changes especially promoter methylation may result in transcriptional gene silencing during cultivation. UCOE can prevent expression instability through reduction in DNA methylation and heterochromatin formation at the site of transgene integration [17, 19]. Consequently, we monitored the antibody expression level stability of UCOE and the non-UCOE pools over 4 months, in the absence of selective pressure. In general, antibody production remained more constant in the UCOE pool. Among the three UCOE cell pools, no considerable change in expression levels of CHO-UHL was observed. However, CHO-UHUL and CHO-HUL pools showed expression reduction, but still maintained their antibody production levels 40% higher than the non-UCOE pool. So, in line with previous studies, it was deduced that UCOE could enhance expression stability over long cultivations and CHO-UHL is the best system for stable antibody production.

## Conclusions

In conclusion, the results of the present work demonstrated that incorporation of UCOE in antibody expression plasmid vectors could result in generation of higher and more stable expression levels relative to conventional (non- UCOE) control vectors. Among the three stable UCOE containing pools studied in this work, CHO-UHL was the most suitable system for improved stable antibody production. Hence, it is concluded that HC expression up-regulation provides more optimized expression conditions relative to enhancement of both HC and LC expression. The novel system illustrated here could propose an important alternative to industrial cell line development approaches.

## Abbreviations

A2UCOE: Human HNRPA2B1-CBX3 locus
CHO: Chinese Hamster Ovary
CMV: Cytomegalovirus
ELISA: Enzyme-Linked Immunosorbent Assay
G418: Geneticin
HC: Heavy Chain
LC: Light Chain
LCRs: Locus Control Regions
mAb: Monoclonal Antibodies
MARs: Matrix Attachment Regions
qRT-PCR: Quantitative Real-Time PCR
UCOE: Ubiquitous Chromatin Opening Element
